# Regulatory Effect of Mung Bean Peptide on Prediabetic Mice Induced by High-Fat Diet

**DOI:** 10.3389/fnut.2022.913016

**Published:** 2022-06-09

**Authors:** Lina Li, Yu Tian, Shu Zhang, Yuchao Feng, Haoyu Wang, Xiaoyu Cheng, Yantao Ma, Rui Zhang, Changyuan Wang

**Affiliations:** ^1^College of Food, Heilongjiang Bayi Agricultural University, Daqing, China; ^2^Library, Heilongjiang Bayi Agricultural University, Daqing, China

**Keywords:** mung bean peptides (MBPs), prediabetes, gut microbiota, hypoglycaemic, inflammatory reaction, oxidative stress

## Abstract

Dietary supplementation with mung bean peptides (MBPs) has several health benefits. However, the effect of MBPs on prediabetes and gut microbiota imbalance caused by a high-fat diet (HFD) has not been thoroughly studied. In this study, dietary supplementation with MBPs for 5 weeks significantly reduced HFD-induced body weight gain, hyperglycaemia, hyperlipidaemia, insulin resistance, inflammation, and oxidative stress and alleviated liver and kidney damage in mice. In addition, it significantly reversed the HFD-induced gut microbiota imbalance, increased the gut microbial diversity, and decreased the abundance of Firmicutes and Bacteroidetes in prediabetic mice. Furthermore, we identified *Lachnospiraceae_NK4A136* and *Lactobacillus* as important eubacteria with the potential to alleviate the clinical symptoms of prediabetes. According to PICRUSt2 analysis, the changes in intestinal microflora induced by MBPs diet intervention may be related to the downregulation of expression of genes such as *rocR*, *lysX1*, and *grdA* and regulation of seven pathways, including pyruvate, succinic acid, and butyric acid. Moreover, 17 genera with significantly altered levels in the intestine of HFD-fed mice, including *Akkermansia*, *Roseburia*, and *Ruminiclostridium*, were significantly correlated with 26 important differential metabolites, such as D-glutathione, anti-oleic acid, and cucurbitacin. Overall, these results show that MBPs diet intervention plays a key role in the management of HFD-induced prediabetes.

## Introduction

According to the tenth edition of the Global Diabetes Survey released by the International Diabetes Federation on 6 December 2021, one in ten adults (20–79 years old) in the world, 537 million people, were living with diabetes. The number of people diagnosed with diabetes is expected to increase to 643 million by 2030 and 783 million by 2045. In 2021, 6.7 million deaths were attributed to diabetes, and at least 966 billion dollars were spent in the medical sector on diabetes treatment—an increase of 316 percent over the past 15 years. In addition, 541 million adults suffer from impaired glucose tolerance. Impaired glucose tolerance is a typical symptom of prediabetes, in which individual blood glucose levels increase but do not meet the diagnostic criteria for diabetes, representing the intermediate state between normal glucose metabolism and diabetes (1). Prediabetes is often more common than diabetes; 10% of patients with prediabetes develop type II diabetes (T2DM) each year (1). It is estimated that by 2030, more than 470 million people worldwide will have prediabetes. With medical intervention, the blood glucose level of patients with prediabetes can be decreased or even reversed to normal levels, to avoid the development of T2DM (2). At present, the main treatment strategies for T2DM include lifestyle improvement or changes and drug therapy. Lifestyle improvement includes eating a healthy diet with regular moderate exercise. Drug therapy is mainly used for people with high fasting blood glucose levels and severely impaired glucose homoeostasis; drugs such as biguidine, sulphonylureas, glinides, and glucotropin analogues are used (3). Mild cases do not currently receive these levels of medication. Long-term use of hypoglycaemic drugs in critically ill patients not only increases the economic burden of patients but also has side effects on the human body, such as gastrointestinal and liver diseases. Therefore, it is of great significance to find safe and efficient natural bioactive substance adjuvants or alternative drugs for early intervention of prediabetes.

In recent years, numerous studies have shown that food-derived soy peptides play an important role in promoting body weight loss and hypoglycaemia (4). Previous studies have shown that the tetrapeptide Val-His-Val-Val (5), heptapeptide Pro-Pro-His-Met-Gly-Gly-Pro (6), polypeptide Vglycin (3) containing thirty-seven amino acid sequences, and black bean peptides (7) extracted from soybean, spotted bean, pea, and black bean have good hypoglycaemic activity, which helps reduce weight, improve insulin resistance (IR), and effectively alleviate the related symptoms of diabetes. Food-derived soybean peptides have attracted considerable attention because of their good permeability, cell proliferation, low toxicity, and mild side effects (8). Mung bean peptides (MBPs) are potential food-borne peptides isolated from mung beans under this research background. They have anti-inflammatory, antioxidant, hypotensive, hypolipidaemic, and other biological properties. However, there are few reports on the hypoglycaemic activity of MBPs.

Gut microbiota, which participates in glucose and lipid metabolism, are closely related to the health of the host and play an important physiological role in the body balance and health of the host. 16S rDNA sequencing is a high-throughput method used to study bacterial composition in samples, interpret the diversity, richness, and structure of the microbial community, and explore the relationship between the microbial community and the host. It is the most widely used method at present for study intestinal flora (9). An increasing number of studies have shown that gut microbiota may be a new therapeutic target for patients with dyslipidaemia, IR, and other obesity-related diseases (10). Previous studies have shown that dietary components can improve obesity, diabetes, and non-alcoholic fatty liver disease and its complications by regulating intestinal microflora (9); therefore, adjustment of the dietary structure is important for patients with prediabetes. Food-derived soybean peptides can be used as regulators and nutritional agents of the intestinal microflora to produce beneficial metabolites by reshaping microbial communities, thereby affecting intestinal homoeostasis and host health. In our previous study, we found that MBPs could reduce IR and correct glucose and lipid metabolism disorders in high-fat diet (HFD)-induced IR mice. In this study, the hypoglycaemic effect of MBPs was further evaluated based on biochemical index detection and histopathological observation, and the difference in intestinal microflora of prediabetic mice after MBPs dietary intervention was studied using 16S rDNA technology to explore the regulatory effect of MBPs on intestinal microflora imbalance in prediabetic mice induced by HFD. In addition, potential mechanisms were explored to explain the specific beneficial effects of MBPs. The results of this study provide new insights into the design and manufacture of healthy and nutritious diets to prevent diabetes.

## Materials and Methods

### Materials and Reagents

Specifific pathogen-free (SPF) C57BL/6 male mice were purchased from Liaoning Changsheng Biotechnology Co., Ltd. (Liaoning, China), with production licence number SCXK (Liao) 2015- 0001. Standard feed and HFD were purchased from Nantong Trophy Feed Technology Co., Ltd. (Jiangsu, China). Mung bean protein powder was purchased from Shandong, Zhaoyuan, Wenji Food Co., Ltd. (Shandong, China). *P-nitrobenzene*-α-D-glucopyranoside and α-glucosidase were purchased from Shanghai Lanji Technology Development Co., Ltd. (Shanghai, China). ELISA kits for insulin (ml001983-J), C-Peptide (ml063022-J), interleukin (IL)-6 (ml063159-J), tumour necrosis factor alpha (TNF-α; ml002095-J), superoxide dismutase (SOD; ml643059-J), malondialdehyde (MDA; ml826369-J), high-density lipoprotein cholesterol (HDL-C; ml037765-J), low-density lipoprotein cholesterol (LDL-C; ml037825-J), and total cholesterol (TC; ml076635) were provided by Shanghai Enzymatic Biotechnology Co., Ltd. (Shanghai, China). The blood glucose metre was purchased from Roche (Shanghai, China). E.Z.N.A. Stool DNA Kit obtained from Shanghai Enzyme-linked Biotechnology Co., Ltd. (Shanghai, China). All other reagents were analytically pure.

### Preparation and Analysis of Mung Bean Peptides

The MBPs were prepared in our laboratory and amplified using the method of Diao et al. (11). The mung bean protein powder (protein mass fraction 80%) was prepared in a 30 L solution with a 10% substrate mass fraction. The pH value was adjusted to 8 with 4 mol/L NaOH solution, and 2% (substrate mass) alkaline protease (2.4 Au/g) was added and stirred in a water bath at 55°C. The pH value was kept constant with a 4 mol/L NaOH solution. The enzymatic hydrolysis was terminated for 5 h, and the pH value was adjusted to 7. The enzyme was inactivated at 100°C for 10 min while being stirred, and an MBPs mixture was obtained. After filtration and desalting, spray drying was performed. When the outlet temperature for spray drying reached 180°C, the inlet temperature reached 80°C. The prepared MBPs powder was refrigerated for later use.

The molecular weight distribution of the MBPs was determined using RP-HPLC. The instrument used was a Waters 2695 High-Performance Liquid Chromatograph (with a 2487 UV detector and Empower workstation GPC software). The chromatographic column used was a TSKgel 2000 SWXL 300 mm × 7.8 mm. The mobile phase consisted of acetonitrile/water/trifluoroacetic acid, 40/60/0.1 (V/V). Detection was performed under UV radiation at 220 nm. The flow rate was maintained at 0.5 mL/min, and the column temperature was maintained at 30°C.

### Inhibitory Effect of Mung Bean Peptides on α-Glucosidase

Some modifications were made by Wang et al. (12) method. A 50 μL aliquot of α-glucosidase solution (0.5 U/mL) was mixed with 50 μL of MBPs solution (0.5–7 mg/mL) and incubated at 37°C for 10 min. Thereafter, 50 μL pPNG solution (2 mM) was added, and the mixture was further incubated at 37°C for 20 min. A sodium carbonate solution (100 μL, 1 M) was added to stop the reaction, and the absorbance was measured at 405 nm. The rate of α-glucosidase inhibition by MBPs was calculated according to Eq. (1):


α-glucosidaseinhibitionrate(%)=[1-(A-sampleA)control-1

(1) /A]control-2×100

Where A_*sample*_ is the absorbance value of the mixture of MBPs, enzyme, and pPNG; A_*control–*1_ is the absorbance value of the mixture after the buffer solution replaces the enzyme solution; A_*control–*2_ is the absorbance value of the buffer solution replacing the sample solution.

### Animals and Experimental Design

Male SPF C57BL/6-mice, aged 46 weeks and weighing 18–20 g, were fed. The photoperiod of light and darkness was 12 h, the room temperature was 25 ± 3°C, and the relative humidity was 50 ± 15%. All mouse experiments were conducted strictly in accordance with the National Laboratory Animal Management Regulations and approved by the Experimental Center of Heilongjiang Bayi Agricultural University. After 1 week of adaptive feeding, the mice were randomly divided into two groups (*n* = 20): the NCD group (fed a normal diet) and the HFD group (fed an HFD). After 5 weeks of feeding, the mice were weighed, the fasting blood glucose (FBG) level was measured, and an intraperitoneal glucose tolerance test was performed to determine whether the model was successfully established. After successful modelling, a 5-week dietary intervention with MBPs was performed in mice that were divided into the following groups: the NCD group (fed with normal diet), NCD + MBPs group (fed with normal diet + MBPs), HFD group (fed with HFD), and HFD + MBPs group (fed with HFD + MBPs). MBPs were added to the drinking water, taking the way of free eating dietary supplement, and add a concentration of 245 mg/kg (13). Weekly body weight, daily feed intake, and MBPs water consumption were recorded. At the end of the experiment, all the mice have fasted for 12 h. The animals were anaesthetised with pentobarbital sodium, and the liver, kidney, and small intestine, as well as their contents, were collected.


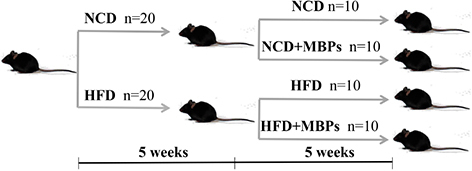


### Fasting Blood Glucose Test

After successful modelling, the FBG level of mice was measured once a week after the dietary intervention with MBPs for 1 week. A small incision was made at the end of the mice’s tail with a surgical blade, and blood samples were collected to check blood sugar levels using a glucose metre. After disinfection with iodophor, mice were returned to their cages. Attention was paid to preventing infection or tail breakage. When the FBG levels of mice exceeded 11.1 mmol/L, it could be concluded that T2DM had been induced (3).

### Detection of Liver Biochemical Indices in Mice

The liver tissues were homogenised in a mixture of chloroform/methanol (2:1, V/V), centrifuged for 10 min at 4°C, 5,000 r/min, and the supernatant was collected. The contents of insulin, C-Peptide, IL-6, TNF-α, SOD, MDA, TC, HDL-C, and LDL-C in the liver were detected following the specific instructions.

### Pathological Observation of Liver and Kidney

We used H&E staining to observe pathological changes in the liver and kidneys of each group. Fresh liver and kidney tissues were fixed with 4% paraformaldehyde for 48 h. Paraffin-embedded sections were stained with H&E and observed under a microscope (200×) (9).

### Gut Microbiota Analysis

Using the E.Z.N.A. Stool DNA Kit, DNA was extracted from faecal samples, and the quality of DNA extraction was detected by agarose gel electrophoresis. DNA was quantified using a UV spectrophotometer. Amplified fragment V3–V4 region, primer sequence: 341F (5′-CCTACGGNGGCWGCAG-3′); 805R (5′-GACTACHVGGTATCTAATCC-3′) (14). PCR products were purified using AMPure XT beads (Beckman Coulter Genomics, Danvers, MA, United States) and quantified using Qubit (Invitrogen, United States). An amplicon pool was used for sequencing. The size and number of amplicon libraries were evaluated using the library quantitative kits of Agilent 2100 (Agilent, United States) and Illumina (Kappa Bioscience, Woburn, MA, United States), respectively. Sorting libraries on the NovaSeq PE250 platform.

The samples were sequenced on the Illumina NovaSeq platform, according to the manufacturer’s recommendations. The paired-end sequences were assigned to the sample according to the unique barcode of the sample, and the barcode and primer sequences introduced into the library were removed. FLASH merges were used to read. According to fqtrim (v0.94), the original read data are filtered under specific conditions to obtain high-quality clean labels. The chimeric sequences were filtered using VSEARCH software (v2.3.4). DADA2 was used to demodulate feature tables and sequences. Diversity was calculated by normalising to the same random sequence. Subsequently, according to the SILVA (release 132) classifier, the characteristic abundance was normalised to the relative abundance of each sample. Alpha and beta diversity was calculated using QIIME 2, R package rendering. BLAST was used for sequence alignment, and each representative sequence was annotated using the SILVA database. The other graphs were implemented using the R package (v3.5.2).

### Statistical Analysis

The experimental results are expressed as mean ± standard deviation (x ± s). SPSS 19 software was used for the analysis of variance. Single-factor ANOVA followed by the *t*-test was used to compare differences between the groups. Pearson’s correlation analysis was used to compare correlations. The Tukey’s HSD test or the Kruskal–Wallis test was used to analyse differences in species diversity between groups. *P* < 0.01 and *P* < 0.05 indicated significant difference between the groups at different levels. *Represented that compared with the NCD group, *P* < 0.05; ^**^Indicated that compared with the NCD group, *P* < 0.01. #Indicated that compared with the HFD group, *P* < 0.05; ##Represents *P* < 0.01 compared with the HFD group.

## Results

### Preparation and Molecular Weight Distribution of Mung Bean Peptides

**FIGURE 1:**
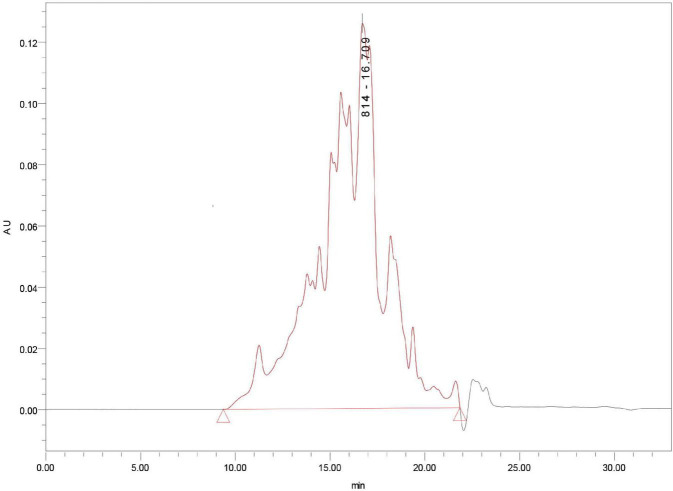
The molecular weight distribution of MBPs.

Mung bean peptides powder was hydrolysed using alkaline protease (2.4 AU/g) for 5 h, and the degree of hydrolysis was 26.42%. MBPs powder was prepared by spray drying, and the molecular weight distribution of the prepared MBPs was determined. As shown in Figure 1, 10,000 Da accounted for 5.54% of the MBPs prepared using this method. The α-glucosidase inhibition rate and IC50 value of MBPs with different molecular weights after ultrafiltration are shown in Table 1. The α-glucosidase inhibition rates of MBPs with different molecular weights were different, and the α-glucosidase inhibition rate of MBPs of <1000 Da was the best. MBPs >5000 Da also showed α-glucosidase inhibitory effects. These data indicate that the mixture of MBPs had a good hypoglycaemic effect.

**TABLE 1:** Results of fractional enrichment of MBPs.

Molecular weight (Da)	Peptide content (g/L)	Inhibition rate of α -glucosidase (%)	IC50 (g/L)
>5000	7.31 ± 0.53	35.60 ± 1.75	10.36 ± 0.63
3000–5000	2.11 ± 0.19	19.37 ± 0.89	8.51 ± 0.47
1000–3000	1.35 ± 0.11	35.06 ± 1.46	1.93 ± 0.08
<1000	0.41 ± 0.07	42.51 ± 2.03	0.48 ± 0.03

### Effects of Mung Bean Peptides on Fasting Blood Glucose of Prediabetic Mice

As shown in Table 2, at the end of modelling, the average FBG value of the NCD group was 6.41 ± 1.16 mmol/L (*n* = 20) and that of the HFD group was 9.62 ± 1.87 mmol/L (*n* = 20). The difference in blood glucose levels between the two groups was significant (*P* < 0.05). The FBG values of the mice in the HFD group reached the prediabetes standard. Throughout the dietary intervention with MBPs, compared with the NCD group, the NCD + MBPs group had a slightly decreased FBG value; however, the difference was not significant. After 5 weeks of MBPs dietary intervention, the FBG value of HFD group mice reached 11.23 ± 2.09 mmol/L (*n* = 10), indicating that some mice had developed T2DM. When the FBG levels exceeded 11.1 mmol/L in mice, it could be concluded that T2DM had been induced (3). The FBG value of the HFD + MBPs group was corrected, and the FBG value of some mice returned to a relatively normal level. The results showed that dietary intervention with MBPs effectively controlled blood glucose and prevented the development of T2DM in prediabetic mice.

**TABLE 2:** FBG of mice.

	FBG (mmol/L)				
	NCD	NCD + MBPs	HFD	HFD + MBPs
At the end of the moulding	6.52 ± 1.39	6.30 ± 0.92	8.30 ± 1.37*	10.94 ± 2.35
Dietary MBPs for 2 weeks	6.64 ± 1.44	6.44 ± 1.20	10.10 ± 2.83*	8.76 ± 1.02
Dietary MBPs for 3 weeks	5.96 ± 0.74	6.67 ± 0.59	10.34 ± 1.34*	8.14 ± 1.08#
Dietary MBPs for 4 weeks	5.53 ± 0.77	5.52 ± 1.51	10.50 ± 1.09*	7.67 ± 1.79#
Dietary MBPs for 5 weeks	6.58 ± 0.62	6.13 ± 1.38	11.23 ± 2.09*	7.43 ± 1.07#

*P < 0.05, NCD compared with HFD mice.

*#P < 0.05, HFD compared with HFD + MBPs mice.*

*Data were presented as the mean ± SD (n = 10).*

### Effects of Mung Bean Peptides on Body and Liver Weights of Prediabetic Mice

As shown in Table 3, compared with a normal diet, HFD can lead to obesity in mice. After MBPs diet intervention, there was no significant difference in body weight between the NCD and NCD + MBPs groups. The bodyweight of the HFD + MBPs group was significantly lower than that of the HFD group (*P* < 0.05), but the difference was not significant. This result indicated that MBPs had a good weight reduction effect in prediabetic mice and were not attributed to the decrease in food intake. As shown in Table 4, after the MBPs dietary intervention, there was no significant difference in liver weight between the NCD and NCD + MBPs groups. The liver weight of the HFD + MBPs group was 14.76% lower than that of the HFD group (*P* < 0.05). This finding showed that MBPs had a repair effect on the liver of prediabetic mice.

**TABLE 3:** Bodyweight of mice.

	Bodyweight (g)				
	NCD	NCD + MBPs	HFD	HFD + MBPs
Primaeval	19.525 ± 0.649	19.918 ± 0.840	19.227 ± 0.460*	19.468 ± 0.632#
1 week	21.268 ± 0.712	21.836 ± 0.817	23.229 ± 1.052*	23.294 ± 1.021
2 weeks	22.078 ± 0.992	22.592 ± 0.879	24.905 ± 1.275*	25.154 ± 1.160
3 weeks	22.583 ± 1.042	22.983 ± 0.802	26.190 ± 1.723*	26.432 ± 1.494
4 weeks	23.722 ± 1.168	24.423 ± 1.139	28.052 ± 3.037*	28.953 ± 1.853
5 weeks	23.948 ± 1.636	24.670 ± 1.147	28.088 ± 3.407*	29.578 ± 2.202
6 weeks	24.351 ± 2.266	24.125 ± 1.120	29.367 ± 2.796*	28.877 ± 2.751
7 weeks	25.266 ± 2.034	24.755 ± 1.507	30.024 ± 2.515*	29.667 ± 3.172
8 weeks	25.199 ± 1.908	24.729 ± 1.570	30.945 ± 2.713*	29.808 ± 2.993
9 weeks	25.548 ± 1.730	25.035 ± 1.883	31.624 ± 2.434*	29.297 ± 3.209#
10 weeks	25.582 ± 1.777	26.241 ± 2.007	32.918 ± 2.463*	29.876 ± 4.382#

*P < 0.05, NCD compared with HFD mice.

*#P < 0.05, HFD compared with HFD + MBPs mice.*

*Data were presented as the mean ± SD (n = 10).*

**TABLE 4:** Weight of mouse liver.

	NCD	NCD + MBPs	HFD	HFD + MBPs
Liver weight (g)	1.0275 ± 0.1047	1.0846 ± 0.0700	1.3082 ± 0.0873*	1.1151 ± 0.1441#

*P < 0.05, NCD compared with HFD mice.

*#P < 0.05, HFD compared with HFD + MBPs mice.*

*Data were presented as the mean ± SD (n = 10).*

### Effects of Mung Bean Peptides on Liver Biochemical Indices of Prediabetic Mice

**FIGURE 2:**
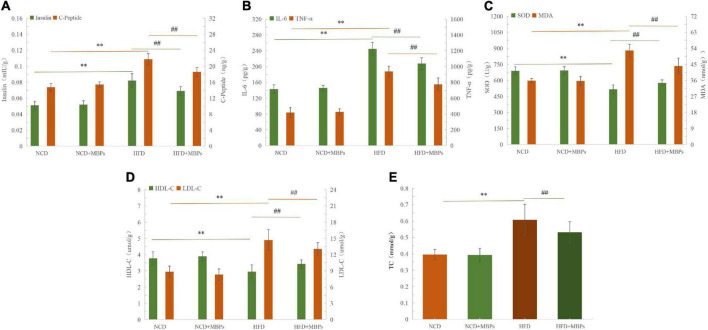
The results of liver biochemical indexes in mice. Effects of MBPs on **(A)** insulin levels and C-Peptide levels, **(B)** IL-6 levels and TNF-α levels, **(C)** SOD levels and MDA levels, **(D)** HDL-C levels, and LDL-C levels, and **(E)** TC levels. Data were presented as the mean ± SD (*n* = 6). **P < 0.01, NCD compared with HFD mice. ##*P* < 0.01, HFD compared with HFD + MBPs mice.

Figures 2A–E show that the levels of insulin, C-Peptide, IL-6, TNF-α, MDA, LDL-C, and TC were significantly increased (*P* < 0.01), and the levels of SOD and HDL-C were significantly decreased (*P* < 0.01) in the livers of mice in the HFD group compared with the NCD group. HFD can lead to abnormal liver biochemical indices—for example, IL-6, TNF-α, SOD, and MDA levels were found to be abnormal. This could induce inflammation and oxidative stress in mice. After 5 weeks of MBPs diet intervention, the levels of insulin, C-Peptide, IL-6, TNF-α, SOD, MDA, HDL-C, LDL-C, and TC in the liver of the NCD + MBPs group were not significantly different compared with the NCD group, indicating that MBPs had no significant effect on liver biochemical indices of NCD mice. The levels of insulin, C-Peptide, IL-6, TNF-α, MDA, LDL-C, and TC were significantly decreased (*P* < 0.01), and those of SOD and HDL-C were significantly increased (*P* < 0.01) in the livers of mice in the HFD + MBPs group compared with the HFD group. The results showed that after MBPs dietary intervention in mice, islet function and blood lipid levels were improved, systemic inflammation and oxidative stress were controlled, and the clinical symptoms of prediabetes were alleviated. It was further proven that MBPs have good hypoglycaemic, lipid-lowering, anti-inflammatory, and antioxidant activities.

### Effects of Mung Bean Peptides on Liver and Kidney of Prediabetic Mice

**FIGURE 3:**
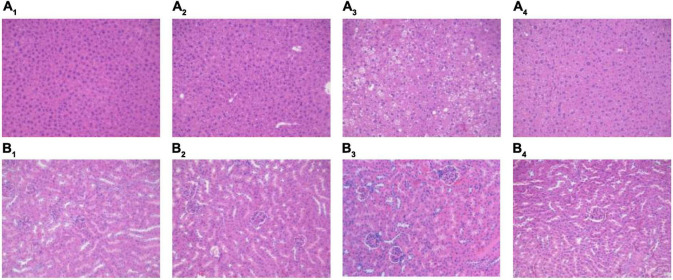
H&E staining results of mouse liver and kidney. Effects of MBPs on **(A_1_)** liver of NCD group, **(A_2_)** liver of NCD + MBPs group, **(A_3_)** liver of HFD group, **(A_4_)** liver of HFD + MBPs group, **(B_1_)** kidney of NCD group, **(B_2_)** kidney of NCD + MBPs group, **(B_3_)** kidney of HFD group, and **(B_4_)** kidney of HFD + MBPs group. Magnification, 200×.

The H&E staining results for the liver and kidney are shown in Figure 3. The overall structure of the liver tissue in the NCD and NCD + MBPs groups was basically normal; the liver cell structure was full, and the hepatic sinus was radially arranged (Figures 3A1,A2). In the HFD group, the overall structure of the liver tissue was abnormal; the liver cell structure was loose, with predominant oedema. Many liver cells showed clear lipid droplets (Figure 3A3). The overall structure of the liver tissue in the HFD + MBPs group was slightly abnormal; the structure of liver cells was loose, with mild oedema (Figure 3A4).

The overall structure of renal tissue in the NCD and NCD + MBPs groups was normal, and the glomerular structure was clear without obvious atrophy, necrosis, or other degeneration. There was no obvious oedema, loss, or necrosis in the renal tubular epithelial cells or expansion of tubular type or other degeneration (Figures 3B1,B2). In the HFD group, the overall structure of the kidney tissue was abnormal, and the glomerular structure was clear without obvious atrophy and necrosis. The loose oedema of the renal tubular epithelial cells was accompanied by fatty degeneration and a small proportion of inflammatory cell infiltration (Figure 3B3). In the HFD + MBPs group, the overall structure of the kidney tissue was mildly abnormal, and the glomerular structure was clear without obvious atrophy and necrosis. A small proportion of renal tubular epithelial cells was loose and oedematous, and no obvious inflammatory cell infiltration was observed (Figure 3B4).

The experimental results showed that the liver and kidney of HFD-induced prediabetic mice showed pathological changes compared with normal mice. MBPs had no significant effect on the liver and kidney of normal diet mice but could alleviate the oedema of liver cells and reduce liver fat content in prediabetic mice. In addition, MBPs alleviated oedema of renal tubular epithelial cells and reduce inflammation. This result indicated that MBPs had a repair effect on the liver and kidneys of the prediabetic mice.

### Effects of Mung Bean Peptides on the Gut Microbiota of Prediabetic Mice

#### Species Diversity Analysis

**FIGURE 4:**
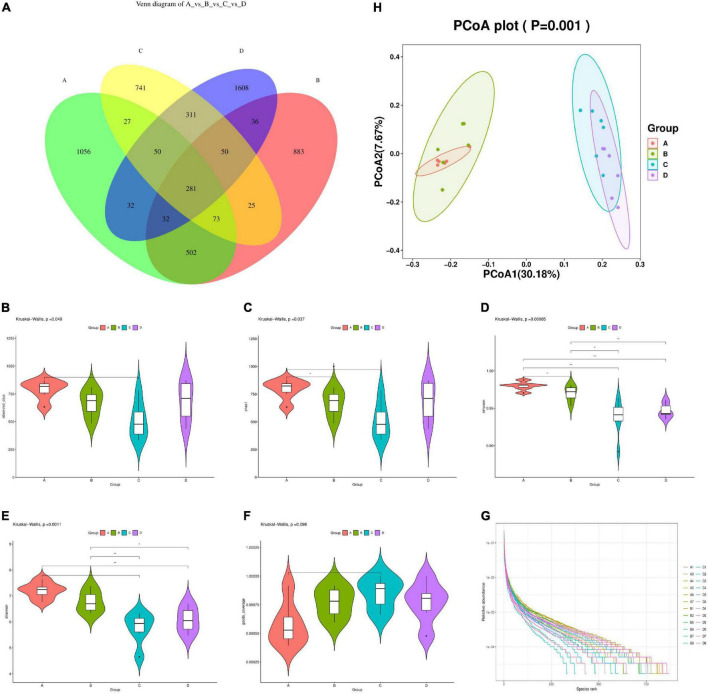
Diversity analysis of intestinal microflora in mice (*n* = 6). **(A)** Venn diagram (NCD vs. NCD + MBPs vs. HFD vs. HFD + MBPs). **(B)** Alpha diversity violin observed OTUs. **(C)** Alpha diversity violin Chao1. **(D)** Alpha diversity violin Simpson. **(E)** Alpha diversity violin Shannon. **(F)** Alpha diversity violin goods coverage. **(G)** Rank abundance graph. **(H)** Unweighted UniFrac PCoA. In the figure, A, B, C, and D represent NCD, NCD + MBPs, HFD, and HFD + MBPs, respectively.

A total of 1,841,018 valid data were detected from 24 samples of four groups, and 6,706 OTUs sequences with similarities above 97% were obtained. The sequencing data in this study were large enough to reflect the majority of microbial diversity information in samples, and the data obtained could be used for subsequent scientific and effective analysis. As shown in Figure 4A, the number of OTUs in the intestinal flora of mice in the HFD group decreased by 24.11% compared to the NCD group. After MBPs dietary intervention, the number of OTUs in the intestinal flora of mice in the HFD + MBPs group increased to 2,400. This result indicated that the diversity of intestinal microflora in HFD-induced prediabetic mice decreased significantly compared to the NCD group. MBPs dietary intervention changed the number of OTUs in prediabetic mice and increased the diversity of intestinal flora. This result indicated that dietary intervention with MBPs improved the diversity of the intestinal microflora in prediabetic mice.

Alpha diversity analysis was used to study the species richness, evenness, and sequencing depth in specific environments. As shown in Figures 4B–F, the number of observed features and Chao1, Simpson, and Shannon indices of the HFD group were significantly decreased (*P* < 0.05), and the good coverage was significantly increased (*P* < 0.05) compared with the NCD group. This result indicated that the species richness, diversity, and evenness of the samples decreased, and the low abundance feature-coating rate of the samples increased in the HFD group compared with the NCD group, indicating that HFD reduced alpha diversity. Compared with the HFD group, the HFD + MBPs group showed an increase in the number of observed features and Chao1, Simpson, and Shannon indices and a decrease in the good coverage. This result indicated that in the HFD + MBPs group, the species richness and evenness of the samples increased and the low abundance feature-coating coverage rate of the samples decreased, indicating that MBPs dietary intervention increased the alpha diversity. The curve of each group of samples tended to be flat; additionally, the sequencing data amount was saturated, indicating that it was gradually reasonable, and more data would yield only a small number of new species/eigenvalues (Figure 4G).

Beta diversity analysis was used to study the species diversity among different environmental communities. As shown in Figure 4H, PCoA analysis revealed that the aggregation of each repeated sample in the NCD group was better than that in the other three groups, indicating that although the intervention effects of HFD and MBPs showed individual differences among different mice, they had an impact on the intestinal flora composition of mice in different groups. The distance between the NCD group and the HFD group was large, indicating that the HFD had a significant effect on the composition of intestinal flora in mice, and the composition of intestinal flora in the two groups was different. The samples in the NCD group were not separated compared with the NCD + MBPs group, indicating that MBPs had little effect on the intestinal flora composition of mice fed common feed. Compared to the HFD + MBPs group, the HFD group had a good separation effect among multiple arrays. This finding indicated that the MBPs dietary intervention changed the intestinal flora composition of prediabetic mice.

#### Species Composition Analysis

**FIGURE 5:**
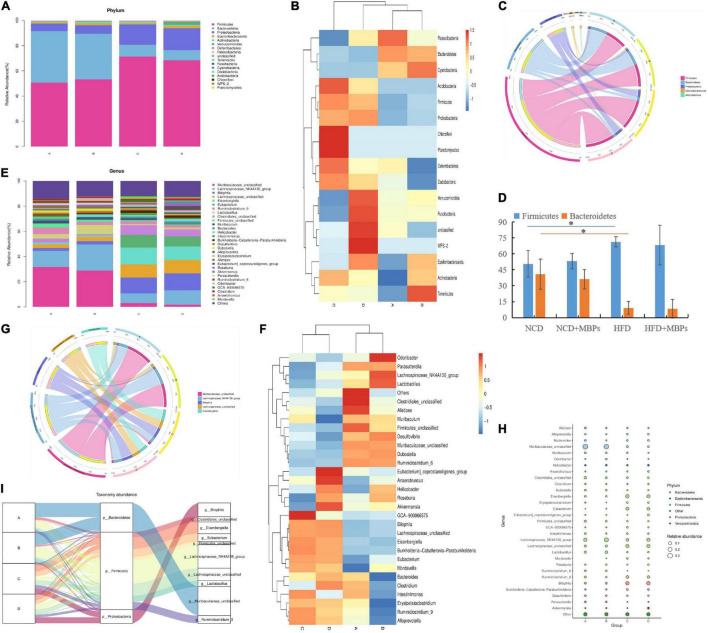
Analysis of intestinal flora species in mice (*n* = 6). **(A)** Phylum abundance group stacked bar. **(B)** Phylum abundance group heatmap. **(C)** Phylum circos top 5. **(D)** The abundance of Firmicutes and Bacteroidetes in each group. **(E)** Genus abundance group stacked bar. **(F)** Genus abundance group heatmap. **(G)** Genus circos top 5. **(H)** Bubble plot. **(I)** Sankey genus abundance phylum. In the figure, A, B, C, and D represent NCD, NCD + MBPs, HFD, and HFD + MBPs, respectively. *P < 0.05, NCD compared with HFD.

The top five phyla with relative abundance were Firmicutes, Bacteroidetes, Proteobacteria, Epsilonbacteraeota, and Actinobacteria (Figures 5A–D). The change in the relative abundance of Firmicutes and Bacteroidetes was associated with the transformation of microbial metabolic potential; therefore, the relative abundance of Firmicutes and Bacteroidetes is considered highly significant (15). HFD could lead to a significant increase in the relative abundance of Firmicutes (*P* < 0.05), a significant decrease in the relative abundance of Bacteroidetes (*P* < 0.05), and an increase in the Firmicutes:Bacteroidetes ratio (*P* < 0.05). This result is consistent with findings of previous studies, confirming that the Firmicutes:Bacteroidetes ratio in the intestinal tract of patients with HFD-induced glucose and lipid metabolism disorders is usually significantly higher than that of healthy people (16, 17). After dietary intervention with MBPs, the relative abundances of Firmicutes and Bacteroidetes did not change significantly in both the NCD + MBPs and HFD + MBPs groups, and the difference in the Firmicutes:Bacteroidetes ratio did not reach statistical significance. After the MBPs diet intervention, the abundance of Firmicutes and Bacteroidetes decreased in prediabetic mice. Wang et al. found that the relative abundances of Firmicutes and Bacteroidetes did not change significantly after HFD mice were fed soybean dietary fibre, and the difference in the Firmicutes:Bacteroidetes ratio did not reach statistical significance (18). This finding is consistent with that of the present study. It is also possible that the targets of bean functional components on HFD are mostly concentrated in the Firmicutes gate, which requires further study.

As shown in Figures 5E–G, the top five taxa with relative abundance at the genus level were *Muribaculaceae_unclassified*, *Lachnospiraceae_NK4A136_group*, *Bilophila*, *Lachnospiraceae_unclassified*, and *Eisenbergiella*. The relative abundance of *Lachnospiraceae_NK4A136_group*, *Lactobacillus*, *Roseburia*, and *Akkermansia* in the NCD + MBPs group showed an upward trend, while that of *Ruminiclostridium_9*, *Intestinimonas*, *Alloprevotella*, and *Erysipelatoclostridium* showed a downward trend, compared with the NCD group. The relative abundance of *Ruminiclostridium_9*, *Intestinimonas*, *Alloprevotella*, and *Erysipelatoclostridium* in the HFD group showed an upward trend, while the relative abundance of *Lachnospiraceae_NK4A136_group*, *Lactobacillus*, *Roseburia*, and *Akkermansia* showed a downward trend, compared with the NCD group. The relative abundance of *Lachnospiraceae_NK4A136_group*, *Lactobacillus*, *Roseburia*, and *Akkermansia* in the HFD + MBPs group showed an upward trend, whereas the relative abundance of *Ruminiclostridium_9*, *Intestinimonas*, *Alloprevotella*, and *Erysipelatoclostridium* showed a downward trend, compared with the HFD group. After MBPs dietary intervention, the relative abundance of the *Lachnospiraceae_NK4A136_group* was close to that of the NCD group. This result suggests that MBPs may regulate the abundance of *Lachnospiraceae_NK4A136_group*, *Lactobacillus*, *Roseburia*, *Akkermansia*, and *Ruminiclostridium* after dietary intervention. Zhao et al. found that adzuki bean could increase the abundance of *Lachnospiraceae_NK4A136_group* and restore the levels of HFD-dependent *Ruminiclostridium_9* (19). Zhang et al. found a significant increase in the abundance of *Lachnospiraceae* in patients with obesity with non-alcoholic fatty liver disease and T2DM (20). Reportedly, the HFD is related to the content of *Lactobacillus*, which can be increased by apple polysaccharide (AP) treatment (15, 21). Studies have also shown that intestinal *Lactobacillus* levels are closely related to impaired glucose tolerance in T2DM patients (22). Zhao et al. found that after the intervention of fermented celery juice in HFD mice, the content of *Lactobacillus* increased, and the contents of *Alloprevotella* and *Ruminiclostridium_9* decreased (23). Wang et al. have found that *Akkermansia* is a favourable factor for reducing weight, improving blood glucose levels, and alleviating IR (24). Wang et al. (9) and Jo et al. (25) found that HFD can increase the content of *Intestinimonas* and *Erysipelatoclostridium*, which can be restored after dietary intervention. These results are consistent with the results of the present study, indicating that MBPs dietary intervention can alleviate the adverse symptoms of prediabetes caused by HFD by regulating the abundance of beneficial and harmful bacteria.

A total of 18 genera belonging to Firmicutes exhibited changes in abundance, including *Lachnospiraceae_NK4A136_group*, *Eisenbergiella*, *Eubacterium*, *Lachnospiraceae_unclassified*, and *Lactobacillus*. Six genera in Bacteroidetes showed changes in abundance, among which only *Muribaculaceae_unclassified* was identified (Figure 5H). Firmicutes, Bacteroidetes, Proteobacteria, and 10 important genera were the most important taxa affecting the diversity of the intestinal flora in mice (Figure 5I). Combined with the results of the previous analysis, it is speculated that *Lachnospiraceae_NK4A136_group* and *Lactobacillus* may be important intestinal microorganisms to alleviate prediabetes.

### Analysis of Species Indigenous Differences

**FIGURE 6:**
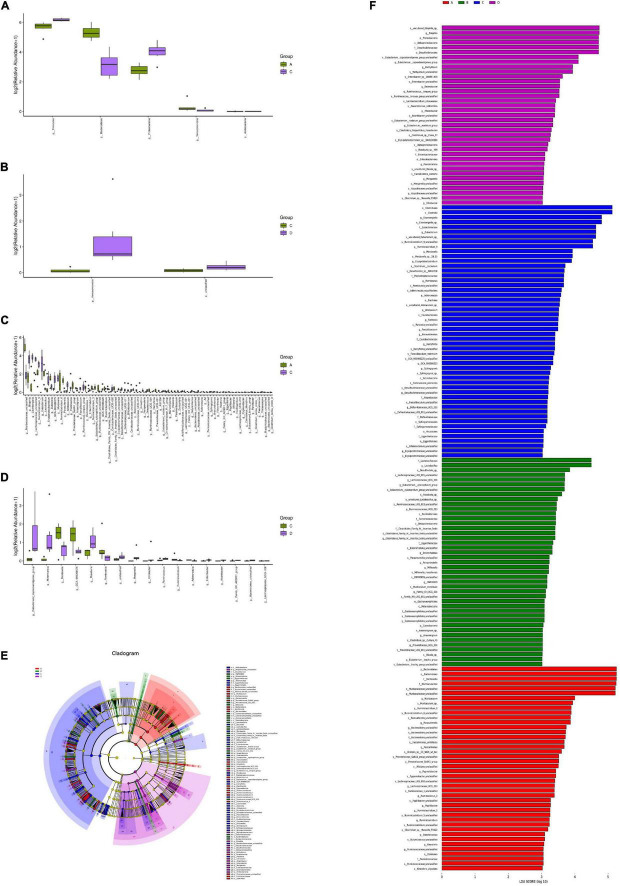
Analysis of significant differences in intestinal flora species in mice (*n* = 6). **(A)** Group A vs. C diff boxplot (phylum). **(B)** Group C vs. D diff boxplot (phylum). **(C)** Group A vs. C diff boxplot (genus). **(D)** Group C vs. D diff boxplot (genus). **(E)** LEfSe group A vs. B vs. C vs. D cladogram. **(F)** LEfSe group A vs. B vs. C vs. D. In the figure, A, B, C, and D represent NCD, NCD + MBPs, HFD, and HFD + MBPs, respectively.

According to the sample species abundance table, the Fisher’s exact test, Mann–Whitney *U* test, and Kruskal–Wallis test were used for the species difference test. Based on the *P* values obtained from the above statistical tests, we determined whether there were significant differences in the species among the different groups. *P* < 0.05 was considered significant. Figures 6A–D show the microorganisms with significant differences at the phylum and genus levels. The LDA effect size (LEfSe) method was further used to analyse the effects of HFD and MBPs on the intestinal flora of mice, and the species with significant differences in abundance between the different groups were screened (Figures 6E,F) to determine the specific intestinal microorganisms in each group. The results showed that HFD led to significant changes in the abundance of Firmicutes, Bacteroidetes, Proteobacteria, Verrucomicrobia, and Acidobacteria five mycotas in prediabetic mice (Figure 6A). There were 78 genera with significant differences, including *Muribaculaceae_unclassified*, *Bilophila*, *Eisenbergiella*, *Lachnospiraceae_unclassified*, and *Ruminiclostridium_9* (Figure 6B). After MBPs dietary intervention, there were two phyla, including Verrucomicrobia and unclassified (Figure 6C), and 17 genera, including *Eubacterium_coprostanoligenes*, *Akkermansia*, *Mordavella*, *GCA-900066575*, and *Roseburia*, in the intestinal flora of prediabetic mice (Figure 6D). We speculated that the adverse reactions in HFD-induced prediabetic mice and the regulatory effect of MBPs diet intervention may be related to *Romboutsia*, *Adlercreutzia*, *Bacteroidetes*, and *Ruminiclostridium*.

### Function Prediction by PICRUSt2

**FIGURE 7:**
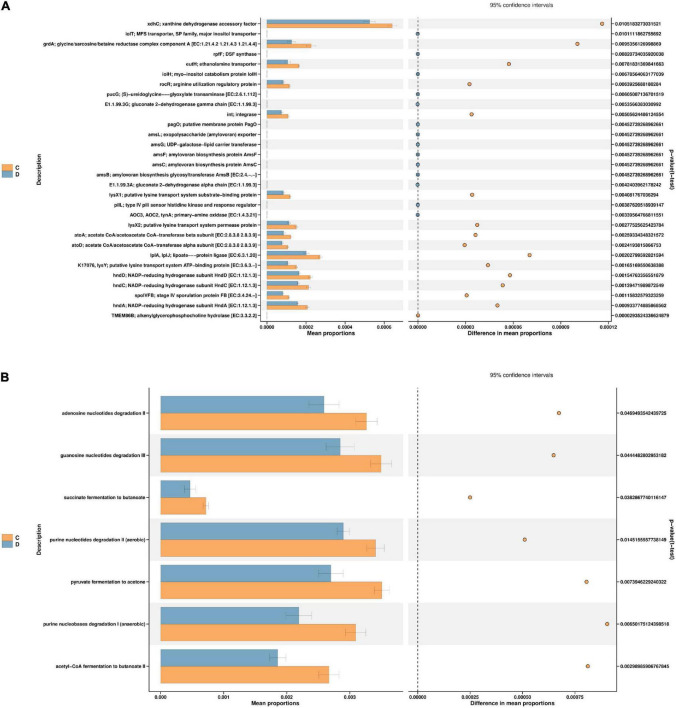
Function prediction (*n* = 6). **(A)** PICRUSt2 KO group C vs. D. **(B)** PICRUSt2 pathways group C vs. D. In the figure, C and D represent HFD and HFD + MBPs, respectively.

According to the *t*-test difference test, the threshold of *P* < 0.05 was used to predict the function of intestinal microorganisms in mice (Figure 7). PICRUSt2 was used to establish a “mapping” between flora and function, and the significant differences in abundance data in the functional database (95% confidence interval) were analysed. It was speculated that the main genes and pathways associated with HFD-induced prediabetes and MBPs diet alleviated the clinical symptoms of prediabetes in mice. As shown in Figure 7A, after the MBPs dietary intervention, the expression levels of 30 genes changed significantly. The main manifestation was the downregulation of xanthine dehydrogenase accessory factor (xdhC), glycine/sarcosine/betaine reductase complex component A [EC: 1.21.4.2 1.21.4.3 1.21.4.4] (grdA), ethanolamine transporter (eutH), arginine utilisation regulatory protein (rocR), integrase (int), putative lysine transport system substrate-binding protein (lysX1), putative lysine transport system permease protein (lysX2), and putative lysine transport system ATP-binding protein [EC: 3.6.3.-] (K17076, lysY), acetate CoA/acetoacetate CoA-transferase beta subunit [EC: 2.8.3.8 2.8.3.9] (atoA), acetate CoA/acetoacetate CoA-transferase alpha subunit [EC: 2.8.3.8 2.8.3.9] (atoD), lipoate-protein ligase [EC: 6.3.1.20] (LplA, LplJ), NADP-reducing hydrogenase subunit (hndA, hndC, hndD), and stage IV sporulation protein FB [EC: 3.4.24.-] (spoIVFB) that affected the microbial composition of the intestinal flora to alleviate the clinical symptoms of prediabetic mice. Previous studies have shown that xdhC is related to nitrogen assimilation, hormone metabolism, and reactive oxygen species production (26). RocR is also involved in regulating arginine utilisation (27). lysX1, lysX2, lysY, and K17076 are involved in the biosynthesis and degradation of lysine (28). LplA and LplJ (29) as well as grdA (30) are involved in glycine metabolism. hndA, hndC, and hndD are involved in the synthesis of lipids, fatty acids, and nucleotides (31). Furthermore, atoA and atoD are involved in the metabolism of short-chain fatty acids (SCFAs) (32). eutH is involved in the metabolism of brain phosphorus and lipid (33). These findings are consistent with those of the present study, further proving that changes in the structure and abundance of intestinal flora after dietary intervention with MBPs can lead to changes in the expression of key genes in the process of glucose and lipid metabolism, thus regulating HFD-induced prediabetes in mice.

As shown in Figure 7B, after dietary intervention with MBPs, changes in gene expression affected several pathways, specifically, the pyruvate fermentation to acetone pathway, succinate fermentation to butanoate pathway, acetyl-CoA fermentation to butanoate II pathway, adenosine nucleotide degradation II pathway, guanosine nucleotide degradation III pathway, purine nucleotide degradation I (anaerobic) pathway, and purine nucleotide degradation II (aerobic) pathway. Cai et al. found that the metabolic function of HFD-induced diabetic mice was inhibited and the function of amino acid metabolism was decreased (34). This finding is consistent with the results of the present study. MBPs dietary intervention can cause changes in amino acid and SCFA metabolism. The results of the PICRUSt2 analysis further support our previous findings, which provide a possible mechanism linking exogenous MBPs intake to the development of prediabetes.

### Relationship Between Intestinal Flora and Biochemical Indices

**FIGURE 8:**
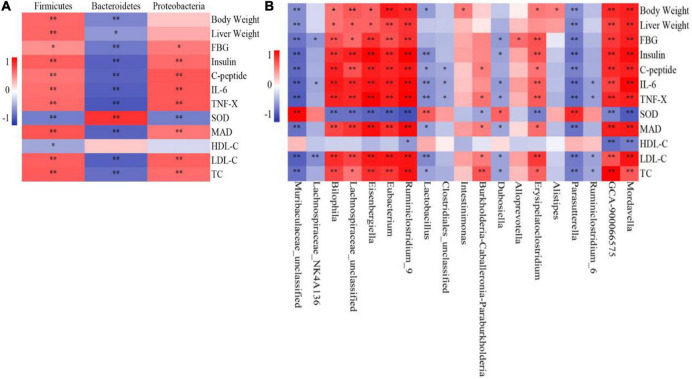
Correlation analysis of intestinal flora and biochemical indices (*n* = 6). **(A)** Phylum, **(B)** Genus. *P < 0.05, **P < 0.01.

The correlation analysis between microbial species and liver biochemical indices in mice showed that Firmicutes abundance was positively correlated with body weight, liver weight, and FBG, insulin, C-Peptide, IL-6, TNF-α, MDA, LDL-C, and TC levels at the phylum level (Figure 8A) and negatively correlated with SOD and HDL-C levels. Proteobacteria abundance was positively correlated with FBG, insulin, C-Peptide, IL-6, TNF-α, MDA, LDL-C, and TC levels and negatively correlated with SOD level but had no correlation with body weight, liver weight, and HDL-C level in mice. Bacteroidetes abundance was negatively correlated with body weight, liver weight, and FBG, insulin, C-Peptide, IL-6, TNF-α, MDA, LDL-C, and TC levels and positively correlated with SOD and HDL-C levels.

At the genus level (Figure 8B), *Bilophila*, *Lachnospiraceae_unclassified*, *Eisenbergiella*, *Eubacterium*, *Ruminiclostridium_9*, *GCA-900066575*, and *Mordavella* were positively correlated with mouse weight, liver weight, and FBG, insulin, C-Peptide, IL-6, TNF-α, MDA, LDL-C, and TC levels and negatively correlated with SOD level. Only *Ruminiclostridium_9*, *GCA-900066575*, and *Mordavella* were negatively correlated with HDL-C levels. *Burkholderia-Caballeronia-Paraburkholderia* was positively correlated with C-Peptide, TNF-α, MDA, LDL-C, and TC levels in mice; negatively correlated with SOD levels; not correlated with other indicators. *Erysipelatoclostridium* was positively correlated with body weight and FBG, insulin, C-Peptide, IL-6, TNF-α, MDA, LDL-C, and TC levels and negatively correlated with SOD levels but not with other indicators. *Intestinimonas* and *Alistipes* were positively correlated with the weight of mice but not with other indicators. *Alloprevotella* was only positively correlated with FBG levels but was not correlated with other indicators.

*Muribaculaceae_unclassified* and *Parasutterella* were negatively correlated with body weight, liver weight, and FBG, insulin, C-Peptide, IL-6, TNF-α, MDA, LDL-C, and TC levels; positively correlated with SOD level; not correlated with HDL-C level. *Lactobacillus* was negatively correlated with body weight and insulin, C-Peptide, IL-6, TNF-α, MDA, LDL-C, and TC levels and positively correlated with SOD levels but not with other indicators. *Dubosiella* was negatively correlated with FBG, insulin, IL-6, TNF-α, MDA, LDL-C, and TC levels and positively correlated with SOD levels but not with other indicators. *Lachnospiraceae_NK4A136* was negatively correlated with FBG, IL-6, and LDL-C levels but was not correlated with other indicators. *Clostridiales_unclassified* was negatively correlated with C-Peptide, IL-6, and TNF-α levels and was not correlated with other indicators. *Ruminiclostridium_6* was negatively correlated with IL-6, TNF-α, LDL-C, and TC levels but was not correlated with other indicators.

Song et al. found that Firmicutes had beneficial effects on body weight and liver TC content, whereas Bacteroidetes was harmful (35). *Lactobacillus* has β-glucosidase activity; it participates in glycoside biotransformation, which can reduce body weight gain (36), and is considered to be a beneficial dominant bacterium (37). *Lactobacillus* and *Akkermansia* are considered helpful in alleviating IR, promoting immune function, and reducing intestinal mucosal permeability and are negatively associated with most glucose and lipid disorders (38). *Parasutterella* was found to be positively correlated with chronic inflammation and lead to IR (39). In HFD-induced T2DM mice, the abundance of *Parasutterella* increased significantly and then decreased after antidiabetic monomer treatment (40). *Lactobacillus* can reduce the TC content and inflammatory factor levels in the serum (41). Based on these findings, we speculated that the selective enrichment or reduction of the abundance of bacteria with positive or negative correlations with physiological biomarkers might be potential targets for MBPs against prediabetes and related metabolic disorders.

### Relationship Between Intestinal Flora and Metabolites

**FIGURE 9:**
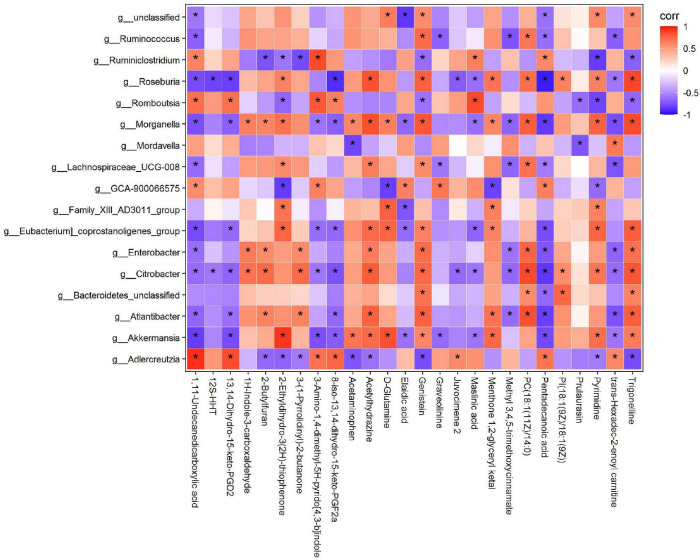
Correlation analysis of intestinal flora and metabolites (*n* = 6). *P < 0.05.

To comprehensively analyse the relationship between the 17 intestinal microorganisms and differential metabolites after dietary intervention with MBPs, a correlation matrix was established based on the Spearman correlation coefficient (Figure 9). Further correlation analysis showed that the key differential metabolite D-glutamine was positively correlated with *Akkermansia*, *[Eubacterium]_coprostanoligenes_group*, *Family_XIII_AD3011_group*, and *Morganella* and negatively correlated with *GCA-900066575*. After MBPs dietary intervention, the content of metabolite 1,11-undecanedicarboxylic acid increased most significantly (*P* < 0.01) and was positively correlated with *Adlercreutzia*, *GCA-900066575*, *Romboutsia*, and *Ruminiclostridium* and negatively correlated with *Akkermansia*, *Atlantibacter*, *Citrobacter*, *Enterobacter*, *[Eubacterium]_coprostanoligenes_group*, *Lachnospiraceae_UCG-008*, *Morganella*, *Roseburia*, and *Ruminococcus*. The content of elaidic acid was positively correlated with *GCA-900066575* and negatively correlated with *Akkermansia*, *Eubacterium_coprostanoligenes_group*, *Family_XIII_AD3011_group*, and *Morganella*. The pentadecanoic acid content was positively correlated with *Adlercreutzia*, *GCA-900066575*, and *Ruminiclostridium* and negatively correlated with *Akkermansia*, *Atlantibacter*, *Bacteroidetes_unclassified*, *Citrobacter*, *Enterobacter*, *[Eubacterium]_coprostanoligenes_group*, *Lachnospiraceae_UCG-008*, *Morganella*, *Roseburia*, and *Ruminococcus*. The prulaurasin content was negatively correlated with *Mordavella* and *Romboutsia*. Trigonelline content was positively correlated with *Akkermansia*, *Atlantibacter*, *Bacteroidetes_unclassified*, *Citrobacter*, *Enterobacter*, *[Eubacterium]_coprostanoligenes_group*, *Morganella*, and *Roseburia* and negatively correlated with *Adlercreutzia*, *Romboutsia*, and *Ruminiclostridium*. *Lachnospiraceae* belongs to Firmicutes and participates in the production of butyrate, an important SCFA that is essential for maintaining intestinal health (42). The expression levels of genes related to metabolic function are closely related to Firmicutes and Bacteroidetes abundance in mice, and some intestinal microorganisms in these two phyla play a balanced role in regulating metabolic homoeostasis (43). These results further confirm that changes in metabolites are related to changes in the intestinal flora. After dietary intervention with MBPs, changes in the structure and content of the intestinal flora led to changes in some important metabolites, thus alleviating the adverse symptoms of prediabetes caused by HFD.

## Discussion

α-glucosidase is a key enzyme required for the regulation of food-derived blood glucose and for the regulation of postprandial blood glucose levels. α-glucosidase inhibitors are a class of compounds that can inhibit the activity of α -glucosidase and slow down the rate of glucose generation by hydrolysed starch in the small intestine and delay glucose absorption, thus reducing postprandial hyperglycaemia. They are used for the treatment of diabetes (44). Studies have shown that bioactive peptides derived from natural products are α -glucosidase inhibitors (6). In this study, we found that mung bean mixed peptide has α-glucosidase inhibitory effects, among which MBPs of <1,000 Da have the best α-glucosidase inhibitory effect (IC50 = 0.48 ± 0.03 g/L). Jiang et al. (44) showed that soybean mixed peptide had good α-glucosidase inhibitory effects, and the spH-C component had the strongest effect (IC50 = 0.27 g/L). Therefore, the MBPs should be further purified to obtain a peptide with higher α-glucosidase inhibitory effects.

Prediabetes refers to the condition of impaired glucose regulation, with blood glucose levels above the normal range but lower than the standard range for diabetes. At present, prediabetes also refers to poor glucose tolerance and/or impaired fasting glucose (1). In this study, the effects of MBPs on prediabetic mice were studied. After HFD induction, the FBG level of the HFD group was 9.62 ± 1.87 mmol/L (*n* = 20), which was higher than the normal FBG level but lower than the standard of 11.1 mmol/L for diabetes. We previously conducted a glucose tolerance test to demonstrate that HFD mice had impaired glucose tolerance and IR (45). Therefore, the modelling of prediabetic mice in this experiment was successful.

At present, most bean proteins or peptides in the market are powdered and consumed by the concoction. Therefore, in this study, mice that were fed MBPs granules were orally administered free food. Regarding the MBPs dose selection, we found in previous experiments that when the concentration of MBPs reached 350 mg/kg/d, inflammatory reactions occurred in the liver of mice. Based on the studies by Jiang et al. (44), Sun et al. (46), and Zhang et al. (13), the final dosage of MBPs used in this experiment was 245 mg/kg/d, which was lower than the safe dose of protein food. As a type of medicine-food homology food, mung bean has multi-component, multi-channel, and multi-target characteristics to alleviate sub-health symptoms, whereas commonly used chemical drugs do not have the above biological effects. Considering these data, a positive control group was not used, which may be one of the limitations of this study.

We previously performed serum biochemical tests in prediabetic mice. Since the liver is a key part of insulin action and a major organ involved in glucose homoeostasis, biochemical tests were performed on the livers of the mice in the present study. Hyperglycaemia, hyperinsulinaemia, hypercholesterolaemia, IR, inflammatory response, and oxidative stress were observed in the HFD-induced diabetic mice. After dietary intervention with MBPs, the above symptoms were relieved, consistent with previous experimental results. HFD feeding has been reported to lead to hepatic steatosis, a preliminary stage of NAFLD, in animal models (47). In this study, H&E staining of liver and kidney in mice showed that the adverse symptoms of liver and kidney in mice were alleviated after MBPs dietary intervention. The experimental results showed that MBPs could enhance the antioxidant defence system of HFD-induced prediabetic mice to inhibit inflammation and reduce oxidative damage due to excessive production of reactive oxygen species metabolites and oxidative damage and lipid peroxidation by pro-inflammatory factors (48). MDA levels not only reflect the degree of lipid peroxidation and indirectly evaluate the degree of cell and tissue damage but also the biochemical link between oxidative stress and inflammation. Inflammatory cells release a large number of active substances at the site of inflammation, resulting in excessive oxidative stress, which in turn activates intracellular signalling cascades to enhance pro-inflammatory gene expression (49). Oxidative stress can cause inflammation, and proinflammatory cytokines can cause both inflammation and oxidative stress (35). In addition, hyperglycaemia can lead to lower levels of antioxidants, thereby weakening the antioxidant defence. Therefore, systemic inflammation and oxidative stress are thought to play key roles in metabolic diseases (50). Sun et al. found that corn peptide could delay the onset of NOD in mice and reduce the incidence of islet inflammation and serum IL-6 content (46). Jingjing et al. (11) and Xie et al. (51) showed that mung bean hydrolysate had good antioxidant activity. The heptapeptides isolated from zebra protein by Ngoh et al. showed significant antidiabetic effects (6).

A continuous increase in blood glucose level can lead to the conversion of glucose to fat, thereby leading to abnormal lipid metabolism (52). An increase in TC and LDL-C levels and a decrease in HDL-C levels can accelerate the development of prediabetes to T2DM, which may further develop into cardiovascular disease. Here, we found that MBPs diet intervention significantly reduced the TC and LDL-C content and increased the HDL-C content in prediabetic mice. Kang et al. found that mung bean ethanol extracts significantly reduced cholesterol levels in KK-Ay diabetic mice (53). Hou et al. found that mung bean supplementation significantly reduced blood lipid levels in hyperlipidaemic mice, including LDL-C and HDL-C (54). Our results are consistent with these results. It has been further suggested that dietary supplementation with MBPs can alleviate hepatic hypercholesterolaemia in prediabetic mice.

The gut microbiota plays an important role in maintaining homoeostasis and alleviating metabolic disorders, and the diversity of the gut microbiota is positively correlated with the host’s ability to resist chronic and immune diseases. A low abundance of gut microbiota is associated with lower efficiency in improving the inflammatory response (23). Changes in gut microbiota, loss of bacterial diversity, and changes in microbial composition have been shown to be closely associated with inflammation and oxidative stress and can be remodelled through interactions between gut microbes and dietary components (55). Many studies have shown that an HFD can reduce the richness and diversity of microbial communities and change their structure. In this study, HFD significantly reduced community richness and diversity, and the MBPs dietary intervention reversed this phenomenon. Furthermore, the intestinal flora structure changed significantly after MBPs dietary intervention. Hou et al. have shown that dietary supplementation with mung bean can effectively restore Chao1 and Shannon indices and thus can effectively prevent the decrease in intestinal microbial community richness caused by HFD; however, the specific functional components are unclear (56). Changes in intestinal microbial composition can affect the development of obesity, IR, and inflammation. Dietary interventions, especially with protein or protein hydrolysates, can alter the composition of gut microbiota and improve the clinical symptoms of prediabetes (57).

At the phylum level, Firmicutes and Bacteroidetes are associated with the two most important categories of chronic intestinal diseases, such as prodromal diabetes. Firmicutes were negatively correlated with body weight, liver weight, and FBG, insulin, C-Peptide, IL-6, TNF-α, MDA, LDL-C, and TC levels and positively correlated with SOD levels. However, Bacteroidetes showed the opposite correlation trend with these indicators. Recent studies have pointed out that abnormalities in the intestinal microflora composition in people with obesity affect many physiological functions related to obesity in mammals, especially the metabolism of SCFAs. Bacteroidetes synthesises the maximum levels of acetic acid and propionic acid, and Firmicutes are the main producer of butyric acid (58). The decrease in the Firmicutes:Bacteroidetes ratio contributes to the regulation of glucose and lipid metabolism and the metabolic mechanism of the inflammatory response (9). Some studies have shown that Firmicutes and Bacteroidetes are positively correlated with obesity (23); however, some Bacteroidetes members are negatively correlated with obesity (24). This issue is currently controversial in academia, and further research is required to test this hypothesis. Our study found that HFD could increase the abundance of Firmicutes, reduce the abundance of Bacteroidetes, and increase the Firmicutes:Bacteroidetes ratio. After the MBPs dietary intervention, the abundance of Firmicutes and Bacteroidetes showed a downward trend, but the difference was not significant. As a result, the Firmicutes:Bacteroidetes ratio did not decrease but increased, consistent with the results of Du et al. (39) and Shen et al. (59). According to existing literature, the first reason for this finding may be the difference in animal models and experimental conditions (60). The second reason may be that the intervention time was too short, and the classification phylum was at a higher level (18). Third, the representative intestinal microflora related to prediabetic mice has not been clarified. Therefore, the intervention time can be appropriately extended in the future, and this mechanism can be studied at a smaller classification level.

At the genus level, the abundance of 19 intestinal microorganisms was significantly correlated with body weight, hormone levels, and liver biochemical indices. This study found that MBPs could increase the abundance of potentially beneficial bacteria related to prediabetes, such as *Lactobacillus* and *Lachnospiraceae_NK4A136_group*, and reduce the abundance of potentially harmful bacteria associated with prediabetes, such as *Ruminiclostridium_9* and *Intestinimonas*. *Lachnospiraceae_NK4A136_group* is a potentially beneficial bacterium that can prevent obesity, and *Akkermansia* is a beneficial bacterium in prediabetes. On the contrary, *Ruminiclostridium* and *Roseburia* are harmful bacteria in prediabetes (39, 50). These data suggest that the intestinal microflora is the microbial target of MBPs and is part of the reason for the treatment of prediabetes.

Identifying changes in serum endogenous metabolites can help us understand the material basis and mechanism that affect the development of prediabetes. Our previous study found that dietary intervention with MBPs could lead to an abnormal amino acid, glycerol phospholipid, fatty acid, alkaloid, and nicotinamide metabolism in HFD-induced prediabetic mice (45). Important differential metabolites included glycine, pyroglutamic acid, D-glutamine, amino adipic acid, and nicotinamide. By analysing the relationship between important differential metabolites and intestinal microbes, we found a correlation between differential metabolites and intestinal microbes, which was consistent with our PICRUSt2 function prediction results. Studies have shown that *Lactobacillus*, a probiotic, can metabolise sugar into lactic acid and participate in the metabolism of Bile Acids (BA). *Anaerotruncus* produces butyric acid, which is associated with obesity (61). *Roseburia* produces SCFAs that are associated with obesity (60). *Akkermansia* is an anti-obesity bacterium that can improve intestinal barrier integrity (62). *Lactobacillus*, *Eisenbergiella*, and *Alistipes* are the main producers of SCFAs (35). The above results are consistent with those of the present study, indicating that MBPs dietary intervention can change the structure and abundance of intestinal flora in HFD-induced prediabetic mice. By regulating the types and numbers of beneficial and harmful bacteria, the expression of key genes in the metabolic process of mice was altered, which further affected the changes in metabolic pathways, leading to changes in metabolites and ultimately affecting the weight, FBG level, glucose tolerance, inflammatory response, oxidative stress response, and cholesterol index of prediabetic mice, demonstrating the role of MBPs in alleviating prediabetes.

Although the mechanism by which MBPs regulate prediabetes is not clear, the results of this study show that MBPs can alleviate HFD-induced prediabetes by regulating intestinal microbial composition and serum metabolite levels. However, it is unclear whether MBPs interfere with metabolism through other pathways; therefore, further analysis at the transcriptome level is required. The key intestinal flora that we screened for regulating prediabetes should be further verified and analysed using qPCR.

## Conclusion

In summary, this study showed that MBPs could improve adverse symptoms, such as weight gain, elevated FBG levels, IR, hypercholesterolaemia, inflammation, oxidative stress, and liver and kidney lesions in HFD-induced diabetic mice. MBPs also reversed the changes in intestinal flora composition and played an important role in the regulation of intestinal flora. These results suggest that the health benefits of MBPs are partly attributed to improvements in intestinal ecological disorders. Our results may provide useful clues for the treatment and prevention of prediabetes; support the further application of MBPs in obesity, prediabetes, T2DM, and NAFLD prevention strategies; and contribute to the utilisation of mung bean by-product resources and development of new therapeutic drugs.

## Data Availability Statement

The original contributions presented in this study are included in the article/supplementary material, further inquiries can be directed to the corresponding author.

## Ethics Statement

The animal study was reviewed and approved by the Animal Experiment Committee of Heilongjiang Bayi Agricultural University.

## Author Contributions

LL: writing – original draft, review and editing, methodology, and data curation. YT: methodology, data curation, and review and editing. SZ and YF: analysis of metabolomics results and proofreading. HW, XC, and YM: methodology and data analysis. RZ: information analysis and literature guarantee. CW: conceptualisation, project administration, resources, funding acquisition, and supervision. All authors have made substantial contributions to the conception and design of the project and critically revised and approved the final submitted version of the manuscript.

## Conflict of Interest

The authors declare that the research was conducted in the absence of any commercial or financial relationships that could be construed as a potential conflict of interest.

## Publisher’s Note

All claims expressed in this article are solely those of the authors and do not necessarily represent those of their affiliated organizations, or those of the publisher, the editors and the reviewers. Any product that may be evaluated in this article, or claim that may be made by its manufacturer, is not guaranteed or endorsed by the publisher.
